# Key Triggers of Osteoclast-Related Diseases and Available Strategies for Targeted Therapies: A Review

**DOI:** 10.3389/fmed.2017.00234

**Published:** 2017-12-20

**Authors:** Haidi Bi, Xing Chen, Song Gao, Xiaolong Yu, Jun Xiao, Bin Zhang, Xuqiang Liu, Min Dai

**Affiliations:** ^1^Department of Orthopaedics, The First Affiliated Hospital of Nanchang University, Artificial Joints Engineering and Technology Research Center of Jiangxi Province, Nanchang, China; ^2^Department of Orthopaedics, The People’s Hospital of Changxing County, Huzhou, China

**Keywords:** osteoclast, osteoporosis, periprosthetic osteolysis, rheumatoid arthritis, Paget’s bonedisease, osteopetrosis

## Abstract

Osteoclasts, the only cells with bone resorption functions *in vivo*, maintain the balance of bone metabolism by cooperating with osteoblasts, which are responsible for bone formation. Excessive activity of osteoclasts causes many diseases such as osteoporosis, periprosthetic osteolysis, bone tumors, and Paget’s disease. In contrast, osteopetrosis results from osteoclast deficiency. Available strategies for combating over-activated osteoclasts and the subsequently induced diseases can be categorized into three approaches: facilitating osteoclast apoptosis, inhibiting osteoclastogenesis, and impairing bone resorption. Bisphosphonates are representative molecules that function by triggering osteoclast apoptosis. New drugs, such as tumor necrosis factor and receptor activator of nuclear factor kappa-B ligand (RANKL) inhibitors (e.g., denosumab) have been developed for targeting the receptor activator of nuclear factor kappa-B /RANKL/osteoprotegerin system or CSF-1/CSF-1R axis, which play critical roles in osteoclast formation. Furthermore, vacuolar (H^+^)-ATPase inhibitors, cathepsin K inhibitors, and glucagon-like peptide 2 impair different stages of the bone resorption process. Recently, significant achievements have been made in this field. The aim of this review is to provide an updated summary of the current progress in research involving osteoclast-related diseases and of the development of targeted inhibitors of osteoclast formation.

## Introduction

The bone tissue in humans is renewed and reconstructed continuously with a dynamic balance between osteoblastic bone formation and osteoclastic bone resorption. Osteoclasts, the only cells with bone resorption function *in vivo*, maintain the balance of bone metabolism by cooperating with osteoblasts that are responsible for bone formation (1). During the process of osteoclast maturation, two hematopoietic factors, macrophage colony-stimulating factor (M-CSF, also called CSF-1) and receptor activator of nuclear factor kappa-B ligand (RANKL), are required (2, 3). Osteoclast differentiation and activation research have focused on tumor necrosis factor (TNF) receptor and TNF-like proteins, such as receptor activator of nuclear factor kappa-B (RANK), RANKL, and osteoprotegerin (OPG). The binding of RANKL to its receptor RANK activates signaling pathways that ultimately lead to osteoclastogenesis; however, this process can be suppressed by OPG, which is a soluble “decoy receptor” for RANKL (4, 5).

Functional disorders of osteoclasts and osteoblasts, particularly those related to the excessive activity of osteoclasts, cause many bone and joint diseases (Table 1) (6, 7). For example, osteoporosis, which occurs in people aged 40 and over and is more commonly found in postmenopausal women, presents as an imbalance in bone resorption and bone formation due to excessive osteoclast activation (8, 9). Excessive activation of osteoclasts induced by released wear particles also leads to periprosthetic osteolysis after artificial joint arthroplasty (10–12). In rheumatoid arthritis (RA), subchondral bone destruction is attributed to excessive bone absorption by osteoclasts after the differentiation and maturation induced by proinflammatory cytokines released by the autoimmune system (13, 14). The mechanism of bone metastases and bone destruction found in cancer is also related to the direct activation of osteoclasts by RANKL, which is secreted by cancer cells (15, 16). In addition, high expression of RANK (the RANKL receptor) on the osteoclast surface is an important factor in Paget’s disease (17). Considering the important roles osteoclasts play in the pathology of the above diseases, agents that modulate aberrant osteoclast differentiation and resorption would be useful in the development of bone-protective therapies (Table 2). Currently, approved and anti-resorptive agents include bisphosphonates (BPs), selective estrogen receptor modulators (SERMs), and monoclonal antibodies against RANKL (e.g., denosumab). Though neutralizing excessive osteoclasts have been partially or mostly mitigated with current therapies, they are far from ideal and still face enormous challenges because of their unexpected adverse effects. The long-term usage of BPs is limited due to the occurrence of severe gastrointestinal reactions, mandible necrosis, and atypical femur fractures (18, 19). In addition, treatment with SERMs is associated with increased risks of stroke and cardiovascular events (20). Finally, while mandible necrosis has been rarely observed in denosumab clinical trials (6 cases among 4,450 patients) (21), its safety and efficacy requires further evaluation. Thus, the identification and development of novel anti-resorptive agents are urgently needed. In-depth studies on new therapeutic targets that inhibit osteoclast formation and bone resorption have made important contributions to treatment and are of great socioeconomic value. The aim of this review is to provide an updated summary of the progress in research involving osteoclast-related diseases and targeted inhibitors.

**Table 1:** Summary of osteoclast-related diseases and targeted inhibitors.

Osteoclast-related bone diseases	Osteoclast formation and function	Critical mechanisms	Current therapies and/or future targets
Osteoporosis	Excessive osteoclast formation and hyperactivated function	Estrogen deficiency, increase in RANKL levels resulting in excessive osteoclast formation and decreased bone formation	Bisphosphonates, calcitonin, estrogen replacement, SERMs, strontiumranelate, PTH peptides, RANKL antibody, sclerostin antibody

Periprosthetic osteolysis	Excessive osteoclast formation and hyperactivated function	Wear particles induce immoderate release of RANKL, resulting in excessive activation of osteoclasts	Bisphosphonates, revision surgery

Rheumatoid arthritis	Excessive osteoclast formation and hyperactivated function	Overexpression of RANKL resulting in excessive activation of osteoclastsMMP-9 and MMP-14 produced by osteoblasts	Immune inhibitors, TNF-α inhibitors, CSF-1R inhibitors, RANKL antibody

Bone tumors	Excessive osteoclast formation and hyperactivated function	Imbalance between RANKL and OPG levels in local bone tissue, resulting in excessive activation of osteoclasts	Bisphosphonates, RANKL antibody

Paget’s bone disease	Excessive osteoclast formation and hyperactivated function	High-RANKL expression leading to osteoclast hyperactivity	RANKL antibody

Osteopetrosis	Impaired osteoclast formation and function	(a) Abnormality in RANKL/RANK/OPG system (b) Mutation of M-CSF factor (c) Mutation of V-ATPase subunit (d) Loss of CLC-7 chloride channels (e) Shortage of cathepsin K (f) Lack of c-Fos protein	Hematopoietic stem cell implantation

**Table 2:** Mechanisms of targeted inhibitors.

Targeted agents	Potential mechanisms	Typical drugs
Bisphosphonates	Inhibit farnesyl pyrophosphate synthase and impair osteoclast polarizing	Pamidronate, risedronate, alendronate, zoledronic acid

RANKL antibody	Specifically bind to and inactivate RANKL	Denosumab

Cathepsin K inhibitors	Removal of cathepsin K action	Odanacatib, balicatib

Sclerostin antibody	Specifically bind to and inactivate sclerostin	Sclerostin antibody

V-ATPase inhibitors	Impair V-ATPase assembly and inhibit osteoclast acidification	Enoxacin, diphyllin, bafilomycin, concanamycin

Glucagon-like peptide 2	Mechanism is still unclear	GLP-2

TNF-α inhibitors	Inhibit TNF-α production and decrease the expression of RANKL and M-CSF	Infliximab, adalimumab, etanercept
Colony-stimulating factor-1 receptor (CSF-1R) inhibitors	Block the binding between CSF-1, IL-34, and CSF-1R	CSF-1R Ab huAB1

### Ethics Statement

This review research was conducted according to the guiding principles of the Ethics Committee of Nanchang University.

### Osteoporosis

Osteoporosis is a systemic skeletal disease characterized by a loss of bone mass and the destruction of bone microstructure, leading to fragility and fractures (22). It is considered to be a multifactorial disease potentially caused by genetic mutations, endocrine disorders, and nutritional deficiency. Hormones, such as estrogen, calcitonin, parathyroid hormone (PTH), and vitamin D, act to maintain the normal bone metabolism (23). PTH functions to improve the production of activated vitamin D and calcium absorption. In contrast, osteoclast activity can be accelerated through the stimulation of PTH, causing further bone resorption. Calcitonin exerts bone-protective effects by transferring calcium into bone tissues when binding to its receptor. In addition, estrogen deficiency reduces the rate of bone remodeling and increases osteoclast formation and resorption (24). Inhibition of the wingless-type and integrase 1 (Wnt) and bone morphogenetic protein signaling pathways, which play critical roles in regulating osteoblast formation, leads to decreased bone formation in postmenopausal women (22). In research on osteoclasts, the RANKL/RANK/OPG system represents an important discovery that has occurred in recent years. RANKL, produced by osteoblasts and bone matrix cells, is the key cytokine that stimulates osteoclast precursor cells to differentiate into mature osteoclasts (7, 25–27). It binds to RANK on the surfaces of osteoclast precursor cells and mature osteoclasts. Through this process, bone resorption is induced by the formation and differentiation of osteoclasts. As a decoy receptor that can block interactions between RANKL and RANK, OPG is also produced by osteoblasts and bone matrix cells. This RANKL/RANK/OPG system plays an important role in the occurrence of osteoporosis (28, 29). Estrogen deficiency is involved in the pathogenesis of osteoporosis in the elderly population, especially in postmenopausal women who are commonly found to have osteoporosis. In postmenopausal women, estrogen deficiency causes a decrease in OPG levels, which leads to an increase in RANKL levels; increased RANKL levels over-activate osteoclasts and result in loss of bone mass (22). These data indicate that the RANKL/RANK/OPG system represents a potential therapeutic target in the prevention and treatment of osteoporosis.

### Periprosthetic Osteolysis

Arthroplasty is a reliable treatment used in cases of severe trauma, end-stage arthritis, and periarticular tumors. Knee and hip arthroplasties are being performed around the world at an increasing rate. However, the long-term use of artificial joints exhibits a major limitation in terms of periprosthetic osteolysis and the loosening induced by the wear particles released from the surface of the prosthesis (30, 31). Although the mechanism of wear particle-induced periprosthetic osteolysis is not clear, it is generally agreed that the excessive activation of osteoclasts caused by wear particles plays a critical role in this process (10–12). As foreign bodies, wear particles can stimulate monocytes/macrophages, fibroblasts, T lymphocytes, etc. to produce large amounts of inflammatory cytokines such as TNF-α, interleukin 1 (IL-1), IL-6, IL-11, IL-17, prostaglandin E2, and monocyte colony-stimulating factor (M-CSF). These inflammatory factors can induce local aseptic inflammation, but can also stimulate osteoblasts to express and release large amounts of RANKL, resulting in excessive activation of osteoclasts and periprosthetic osteolysis (32–34). This periprosthetic osteolysis induces loosening; in time, the instability caused by the loosening may further increase mechanical wear and produce more wear particles, resulting in more severe osteolysis. This creates a vicious cycle between periprosthetic osteolysis and loosening in this pathological process (35). Although the relative motions of the components of artificial joints and material corrosion and degradation *in vivo* during the use of prostheses will inevitably lead to the generation of wear particles, the effective inhibition of osteoclast formation, and bone resorption may be an effective way to prevent the loosening of prostheses and therefore extend their lives.

### Rheumatoid Arthritis

Rheumatoid arthritis is a chronic systemic autoimmune disease characterized by progressive irreversible damage of bone and cartilage. Although the detailed mechanism of bone and cartilage destruction in RA has not yet been elucidated, the formation and increased activity of osteoclasts caused by an imbalance in the ratio of RANKL and OPG is considered to be the main factor responsible. Recent studies have revealed the presence of several mature osteoclasts and osteoclast precursor cells in localized lesions in RA. The overexpression of RANKL by active lymphocytes, macrophages, osteoblasts, etc. leads to excessive proliferation and abnormal activation of osteoclasts caused by the binding of RANKL to RANK on the surface of osteoclast precursor cells and mature osteoclasts. In addition to the overexpression of RANKL in damaged joint bone tissue, *RANKL* mRNA is also expressed by fibroblasts in the synovial tissue, which leads to the production of the RANKL protein (36). Kotake et al. isolated multinucleated cells from the synovial lesions of RA patients and showed that they could form bone absorption pits, thus confirming them to be osteoclasts (36). The formation of bone pits can be inhibited by OPG, and the number of pits formed is closely related to the ratio of *RANKL* and *OPG* at the mRNA level. Therefore, quantitative analysis of the *RANKL/OPG* levels in the synovial tissue and synovial fluid may contribute to the early diagnosis of RA. Moreover, MMP-9 and MMP-14 produced by osteoblasts are also important factors that lead to the degradation of the cartilage matrix, pannus formation, and migration of osteoclasts to the bone surface. All of these factors contribute to the erosion of the articular cartilage, subchondral bone, and synovial surface in RA, where osteoclasts play a key role.

### Bone Tumors

Primary or secondary tumors are commonly found in orthopedics, but the success of clinical therapy for such tumors is limited due to the characteristics of invasion, metastasis, and recurrence. In-depth studies in recent years have shown that the RANKL/RANK/OPG system affects tumor biology by regulating osteoclast activity (37–39), imbalances in RANKL and OPG levels in local bone tissues are the main reason for increases in osteoclast bone resorption (40, 41). A previous study showed that the expression levels of *OPG* and *RANKL* mRNA in giant cell tumors of the bone are much higher than those in normal bone tissues (42, 43). Sezer et al. also studied the expression of RANKL and RANK in biopsy specimens of multiple myeloma (44). Data from the study by Sezer et al. also revealed lower serum OPG levels in multiple myeloma patients compared with those in healthy humans and similar patients without bone destruction (44). Although there is sufficient evidence indicating the effect of the RANKL/RANK/OPG system in bone metastases, the mechanism of metastasis is not entirely clear. However, abnormal osteoclast activation, which is caused by an imbalance in RANKL and OPG levels, is considered to be responsible for most tumors.

### Paget’s Bone Disease

Paget’s disease of the bone is a metabolic bone disease accompanied by increased bone resorption and abnormal bone formation. This results in an increased risk of fracture caused by structural disorder, leading to a decrease in the mechanical properties of the bone (45, 46). Some studies have indicated that high-RANKL expression leading to osteoclast hyperactivity is an important factor in Paget’s disease (47, 48). Roodman (49) and Roodman and Windle (50) also showed that the number of osteoclasts in patients with Paget’s bone disease is increased, the osteoclasts are larger, and the number of nuclei is hundreds of times higher than that in normal cultures. In addition, whether the point of origin of the disease is the bone marrow or peripheral blood, mononuclear cells always exhibit a high degree of sensitivity to RANKL, and differentiation to mature osteoclasts seems to be increased (47).

### Osteopetrosis

Osteopetrosis is a metabolic bone disease characterized by increased bone mass caused by polygenic disorders. Disorders in osteoclast formation and loss of osteoclast function are the main reasons for decreased bone resorption and increased bone mass. Recent studies have suggested that decreased bone resorption could be caused by abnormalities in the RANKL/RANK/OPG system, lack of c-Fos protein, and mutations in M-CSF, while mutations in the vacuolar (H^+^)-ATPase (V-ATPase) subunit, loss of CLC-7 chloride channels, and a shortage of cathepsin K are the most common reasons for osteopetrosis caused by bone resorption disorders. Bone marrow transplantation and the subsequent differentiation of hematopoietic stem cells from the implanted new bone marrow into mature and functioning osteoclasts is a treatment option for osteopetrosis.

## Targeted Osteoclastic Inhibitors

There is a wide spectrum of diseases induced by osteoclast dysfunction, and excessive activation of osteoclasts plays a dominant role in most of these diseases. Therapies to inhibit osteoclast formation and bone resorption efficiently and safely are ideal approaches to combat such diseases. Frequent and long-term clinical use of BPs to reduce osteoclast formation is associated with serious complications including gastrointestinal reactions, mandible necrosis, and non-specific femur fractures (51–53). Monoclonal antibodies against RANKL, such as denosumab, are a new class of drugs used for the targeted inhibition of osteoclast formation. These act by blocking the RANK/RANKL/OPG regulatory system, and this has been a major discovery in the field of osteoblast research in recent years. Glucagon-like peptide 2 (GLP-2), cathepsin K, and V-ATPase inhibitors are also expected to be of use in inhibiting osteoclast formation, and other measures such as anti-TNF-α therapy can also be used.

### Bisphosphonates

Bisphosphonates, such as alendronate and zoledronic acid, are anti-bone resorption drugs commonly used as a therapeutic choice for bone diseases including Paget’s disease of the bone and myeloma. Their ability to inhibit osteoclast resorption is the desired pharmacological effect. McClung (54) and Russell et al. (55) showed that BPs could effectively inhibit bone resorption by binding to hydroxyapatite (HAP) crystals, which results in blocking the prenylation process of proteins due to the inhibition of farnesyl pyrophosphate synthase. When prenylation is blocked, the osteoclast cytoskeleton cannot be rearranged and polarized as an enclosed area for adhering to the bone surface cannot be formed. Thus, although BPs have been confirmed to show an inhibitory effect on osteoclast resorption, this desired clinical effect is often limited by the above-mentioned complications. Due to the high affinity of BPs to HAP crystals in the bone matrix, novel bone-targeting agents have been synthesized based on the molecular structure of BPs. Toro et al. and Rivera et al. demonstrated that BP-enoxacin, a BP derivative, had an inhibitory effect on osteoclast formation and bone resorption and represented an ideal therapeutic agent for preventing orthodontic tooth movement (56, 57). Furthermore, our previous study showed a beneficial effect of BP-enoxacin on cortical bone mass and strength in ovariectomized rats (58). We speculate that it would be an exciting and insightful approach to explore bone-targeting agents that are BP “carriers.”

#### CSF-1/CSF-1R Axis Inhibitors

The binding of M-CSF (also called CSF-1) to its tyrosine kinase receptor (CSF-1R) promotes the differentiation of myeloid progenitors into monocytes, macrophages, dendritic cells, and osteoclasts. *In vivo*, circulating CSF-1 regulates the migration, proliferation, and survival of macrophages, which is beneficial to the innate and adaptive immune system, as well as osteoclastogenesis at multiple levels (59). Theoretically, targeting of the CSF-1/CSF-1R axis to modulate macrophage populations may result in potential therapeutic effects in four types of clinical diseases: inflammatory diseases, cancer, autoimmunity, and bone diseases (60). Antibodies against CSF-1 and its receptor, as well as specific inhibitors of CSF-1R kinase, have been evaluated either in animal models or in patients. As reported, the *in vivo* administration of CSF-1 exacerbated the inflammation and joint erosion in collagen-induced arthritis (61, 62) due to the role of CSF-1 in the pathology of osteoclastogenesis and subsequent osteolysis. Thus, anti-CSF-1 antibody or blockade of CSF-1R reduces inflammation in humans and in RA models (63, 64). According to the research of Cenci et al. (65), CSF-1 facilitates the process of bone loss in ovariectomized mice. In contrast, Gow et al. (66) observed an osteopetrosis phenotype in CSF-1-deficient animals due to the deficient production of bone-resorbing osteoclasts. Therefore, it is expected that anti-CSF-1 may be beneficial in treating human osteoporosis. Furthermore, it was demonstrated by Rietkotter et al. that anti-CSF-1 therapy was beneficial in preventing carcinoma invasion induced by monocyte-derived cells (67). Although an increasing number of studies have indicated the significant role of CSF-1 in osteoclastogenesis and the efficacy of anti-CSF-1/CSF-1R therapy in treating osteoclast-related diseases, further investigations to determine the safety and side effects of these methods still need to be conducted.

Interleukin-34 was first discovered to be a second ligand of CSF-1R in 2008 (68). It was reported that CSF-1 and IL-34 share structural homology and have largely overlapping effects in regulating monocyte survival and osteoclastogenesis (69, 70). Previous evidence had demonstrated that IL-34, both from giant cell tumors and gingival fibroblasts, plays a critical role in RANKL-induced osteoclast formation as a complete substitute for CSF-1 and that the systemic administration of IL-34 would result in a decrease in trabecular bone mass (71, 72). Cheng et al. (73) confirmed this opinion and demonstrated that IL-34 promotes the proliferation and differentiation of bone marrow macrophages by stimulating p-STAT3 expression, as well as inhibiting the expression of Smad7 in the absence of CSF-1. Regretfully, IL-34 has not been clinically tested for the potential existence of other receptors and adverse pathologies mediated by over-activated macrophages.

### Anti-RANKL Monoclonal Antibody (e.g., Denosumab)

The RANKL/RANK/OPG axis is the key regulatory system that decides whether differentiation occurs. RANKL, a member of the TNF superfamily, is produced and secreted by osteoblasts, bone stromal cells, fibroblasts, and activated T cells. The interaction between RANKL and RANK (surface receptors on osteoclast precursor cells) promotes osteoclast differentiation and maturation and helps osteoclasts survive (74, 75). As a pseudo-receptor of RANKL, OPG can also inhibit osteoclast formation and accelerates apoptosis by binding to RANKL, which inhibits the interaction between RANKL and RANK. RANKL is therefore treated as an ideal target for inhibiting osteoclast formation based on the information obtained so far regarding the RANKL/RANK/OPG system. Denosumab, a synthetic IgG2 monoclonal antibody, can also specifically bind to and inactivate RANKL using the same action mechanism as OPG. In 2010, denosumab was approved for use in treating postmenopausal osteoporosis. A phase III trial, conducted over 3 years, has indicated that the incidence of hip and vertebral fracture decreased by 41 and 68% after administration of 60 mg of denosumab every 6 months (76). To evaluate the long-term efficacy and safety of denosumab use for up to 10 years, participants from the Fracture Reduction Evaluation of Denosumab in Osteoporosis every 6 Months (FREEDOM) trial were asked to join the 7-year FREEDOM Extension trial (clinicaltrials.gov: NCT00523341). This trial reported a sustained reduction in bone turnover markers and progressive increase in bone mineral density in the long-term denosumab treatment group, resulting in the maintenance of low-fracture rates (21, 77–79). However, several side effects of denosumab were also observed in this extension trial, including malignancy, eczema/dermatitis, pancreatitis, endocarditis, delayed fracture healing, and infections, especially the occasional occurrence of opportunistic infections, and these should be taken into serious consideration (79).

### Anti-Sclerostin Monoclonal Antibody

Sclerostin is a small protein encoded by the *SOST* gene and produced in osteocytes. It responds to mechanical stress and targets the Wnt signaling cascade. When activated, sclerostin acts as a key negative regulator of bone anabolic metabolism and exhibits an inhibitory effect on osteoblast differentiation and bone formation (80, 81). Patients or gene mutation mice with consistently low levels of sclerostin due to rare skeletal disorders exhibit high-bone mineral density and low-fracture risk (81). Thus, anti-sclerostin therapy could potentially be used to treating bone metabolism diseases resulting in low-bone mass. Recently, humanized anti-sclerostin antibodies, such as romosozumab (AMG785), blosozumab (AMG167), and BSP804, have been synthesized and subjected to clinical trials. Increased bone mass at the spine and hip, along with modified bone turnover markers (increased bone formation markers and decreased resorption markers) have been observed in romosozumab and blosozumab clinical trials (82, 83). Following these trials, research has been conducted to clarify whether the increased bone mineral density resulted in an improvement in bone mechanical properties. Finite element analysis was employed to assess the strength of the spine (L1 vertebral body) and proximal femur under a simulated compression overload. Results showed that both spinal and femoral strength had increased from the baseline (27.3 versus −3.9%, *P* < 0.0001 and 3.6 versus −0.1%, *P* = 0.059, respectively) and that gains in bone mechanical properties corresponded to gains in bone mineral density (84). Additionally, research conducted on animal models or under other conditions indicated a potentially wide therapeutic application of anti-sclerostin antibodies in treating other bone and joint diseases, such as RA, osteoarthritis, and bone complications of type 2 diabetes mellitus (85). The most common side effects included elevated liver enzymes and injection site reactions. However, hypotheses regarding the relationship between sclerostin and cardiovascular events, intracranial pressure, and some tumors require further investigation (23).

### Cathepsin K Inhibitors

Cathepsin K, which is specifically expressed and secreted by activated osteoclasts during bone resorption, is a key enzyme in the degradation of critical proteins in the bone matrix, including type I collagens (86). Bone resorption can be inhibited by the removal of cathepsin K from osteoclasts. Unlike other anti-resorptive drugs, cathepsin K inhibitors do not affect osteoclast activity, and osteogenic activity is maintained by the cross-coupling of osteoblasts to osteoclasts (87). Balicatib has been artificially synthesized as a specific cathepsin K inhibitor. However, phase II clinical trials for balicatib were discontinued because of morphea-like skin changes observed in the participants (88). Another new cathepsin K inhibitor, odanacatib, is orally selective (89). According to a 2-year randomized controlled study, 3.2 and 5.5% increases were observed in the BMD of the hip and spine, respectively, after odanacatib therapy (90). Recent research has indicated that the beneficial effect of odanacatib in improving bone mineral density is dose-dependent and persists for up to 5 years. Along with increased bone mineral density, the risk of fragility fracture was reduced, and the effect was similar to that of BPs and denosumab (91). Aside from its therapeutic efficacy, however, the safety of odanacatib should be studied and seriously evaluated. According to reported investigations, increased risks of stroke, arterial fibrillation, and atypical fractures were observed during the treatment procedures, although these types of agents are still under development (92).

### V-ATPase Inhibitors

During the process of bone matrix degradation, an acidic microenvironment, which is created by V-ATPase that pump protons into the resorption lacuna, is necessary for osteoclast bone resorption. It has been shown that therapeutic interventions that involve modulating osteoclast V-ATPase activity would be reasonable for the treatment of osteoporosis and other osteolytic diseases (93). V-ATPases are protein complexes composed of at least 14 different protein subunits and are responsible for the active transmembrane transport of hydrogen ions *in vivo*. V-ATPases are organized into V1 and V0 domains, which have two different functions. The V1 domain is composed of eight different subunits (A–H), is located in the cytoplasm, and generates energy by ATP hydrolysis. The V0 domain contains six different subunits (a, c, c′, c′′, d, and e) and is involved in the active transmembrane transport of hydrogen ions (94, 95). In addition, the Ac45 and M8-9 auxiliary subunits found in mammal V-ATPases have a collaborative effect in facilitating hydrogen ion transport (96, 97). Some studies have shown that the dysfunction of V-ATPases may lead to the occurrence of many diseases such as osteopetrosis and tumor bone metastasis (98). V-ATPases are thus considered to be a potential target in the treatment of such osteoclast-hyperactive diseases such as osteoporosis and bone tumor metastasis. In 2002, two research groups reported that bafilomycin and concanamycin can inhibit the acidification of V-ATPases by affecting the c protein subunit in the V0 domain (99, 100), whereas diphyllin, as a new V-ATPase inhibitor, inhibits osteoclast resorption and apoptosis (101). In recent years, enoxacin has also been confirmed as a type of V-ATPase inhibitor that acts by blocking the binding of the B2 subunit to actin microfilaments, thus destroying suture zone formation in osteoclasts and inhibiting osteoclast acidification (102). Although there are many V-ATPase inhibitors, none has been subjected to a clinical trial. Thus, the mechanisms, targets, efficacy, and safety of these drugs *in vivo* remain to be studied in future research and feasibility studies.

### Glucagon-Like Peptide 2

Glucagon-like peptide 2, whose secretion has a clear circadian rhythm and is regulated by diet, is a peptide hormone produced by intestinal mucosal cells (103). Henriksen et al. (104) found that bone remodeling also shows a circadian rhythm with a close relationship to food intake and eating time. Osteocalcin is a marker that is closely related to bone formation during the treatment of osteoporosis. Another study by Henriksen et al. (105) indicated that GLP-2 has no influence on bone formation due to the increase in bone resorption and the stable expression of osteocalcin, which occurs after GLP-2 treatment before sleeping. Although some studies have shown that GLP-2 can inhibit bone resorption and increase bone density, its mechanism is unclear, particularly with regard to its influence on the biological function of osteoclasts, which is still poorly understood.

### TNF-α Inhibitors

The release of cytokines is closely related to RA and other bone destructive diseases. TNF-α is stimulated by activated T cells, macrophages, and synovial cells under inflammatory conditions and is the most critical inflammatory cytokine, causing excessive activation of osteoclasts (106, 107). The expression of TNF-α has several effects on osteoclastogenesis. RANKL is secreted is large quantities by osteoblasts and bone stromal cells (108), while the expression of RANK on the surface of osteoclast precursor cells increases and the sensitivity of RANK to RANKL is enhanced due to the recruitment of osteoclasts (109). Furthermore, TNF-α can stimulate the expression of another cytokine, M-CSF, which maintains the continuous formation and survival of osteoclasts (110). Based on the above-mentioned roles in bone destructive diseases, TNF-α therefore represents a candidate therapeutic target. Currently, biological targeted therapy using cytokines can be roughly divided into two categories: monoclonal antibodies and soluble receptors, each with different mechanisms. Monoclonal antibodies can be used as cytokines or cytokine receptors, while soluble receptors can pre-capture and inactivate cytokines before the connection between cytokines and cytokine receptors is established. Infliximab, adalimumab, and etanercept, representing TNF-α antagonists, have been investigated in clinical trials for use in the treatment of RA (111, 112).

## Conclusion

**Figure 1:**
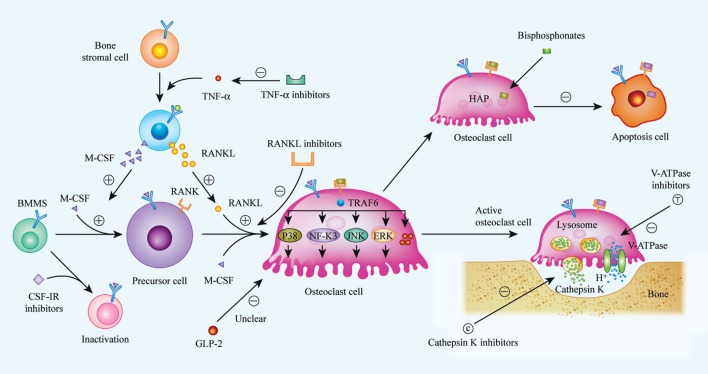
Biological procedures of osteoclast differentiation, bone resorption, and mechanisms of current or future therapeutic drugs. Osteoclasts matured from bone marrow hematopoietic stem cells (BMMs) with the stimulation of two critical factors, M-CSF (CSF-1) and receptor activator of nuclear factor kappa-B ligand (RANKL). When binding to its specific receptors [CSF-1R and receptor activator of nuclear factor kappa-B (RANK)] on BMMs membrane, a series of cascades are activated, and BMMs were then differentiated into matured osteoclast. Realizing the importance of M-CSF and RANKL in osteoclast differentiation, inhibitors to CSF-1R and RANKL were considered as available strategy to suppress over-activated osteoclasts. Bisphosphonates, a widely used anti-osteoporosis agent, can be absorbed by osteoclast and induce osteoclast apoptosis. Additionally, it has been indicated that GLP-2 is a negative regulator of osteoclast differentiation, thus, the exact mechanisms are still unclear. Bone resorption is demonstrated as specific function of osteoclast, and bone matrix degradation is induced by the release of cathepsin K, as well as H^+^, and the release of H^+^ is enabled by V-ATPase on the membrane of matured osteoclast. So that, cathepsin K and V-ATPase are considered as another two targets to impair osteoclast function, especially, inhibitors of cathepsin K, such as Odanacatib, Balicatib are undergoing clinical trials. (⊝ represents inhibitory or down-regulated effect, ⊕ represents facilitated or up-regulated effect).

Osteoclasts are responsible for the necessary function of bone resorption *in vivo*, but the clinical treatment of many diseases caused by osteoclast dysfunction, in particular by excessive activation of osteoclasts, faces enormous challenges. The development of new targeted drugs designed to inhibit osteoclast formation is urgently needed for clinical treatment. According to research on new drugs to inhibit osteoclast formation, the RANKL/RANK/OPG system, CSF-1/CSF-1R axis, cathepsin K, sclerostin, V-ATPases, and the cytokine TNF-α are currently considered as potential critical targets (Figure 1). The broad application prospects of denosumab, anti-sclerostin antibodies, cathepsin K inhibitors, and TNF-α inhibitors are currently being studied in phase II and phase III clinical trials. V-ATPases provide a theoretical advantage due to their important roles in osteoclast acidification and bone matrix degradation and the fact that they have been found to have no effect on osteogenic activity. However, although several V-ATPase inhibitors have been identified, no mature inhibitor has entered clinical trials because of limitations such as the associated toxicity, an unclear mechanism of action, and the lack of credible animal research models. GLP-2, a polypeptide produced by the body, has no physiological side effects and can regulate the circadian rhythm of bone remodeling and shorten bone resorption time, which indirectly prolongs osteogenic time. However, few studies have been conducted on the relationship between GLP-2 and bone metabolism, and the mechanism of action of GLP-2 remains unclear; therefore, its conversion and clinical application requires further research and feasibility studies.

Taken together, we propose that future research would be best served by focusing on two aspects. First, more studies should be conducted to explore and clarify the underlying mechanisms of each disease. Although, it has been reported that osteoclasts are the key factor triggering osteolytic diseases, the influence of osteoblast formation and activity should also be considered, as cross-talk between osteoclasts and osteoblasts exists in bone metabolic processes. Second, randomized, multicenter, controlled, and long-term studies are urgently needed to confirm the safety and efficacy of newly developed pharmacological agents. Anti-sclerostin antibody, which is associated with bone formation, increased bone mineral density, and suppressed bone resorption, is distinct from other anabolic agents and shows the potential for widespread application in treating diseases associated with aberrant bone metabolism. In addition, V-ATPases consist of many subunits, most of which are considered suitable targets for the development of novel agents, and the modulation of V-ATPases theoretically should not affect the formation of osteoclasts. Possibly, it would be the original source in drug discovery. Osteoporosis is the most prevalent bone metabolism disease, especially among postmenopausal women. Currently, developed new anti-osteoporosis drugs, such as denosumab, are clearly able to modify osteoporotic bone quality, enhance bone mechanical properties, and subsequently reduce the risk of fragility fractures due to accelerated bone turnover. There are high expectations for these newly discovered drugs, although most are still under development or awaiting approval.

## Author Contributions

XL, MD, and BZ designed most of this review. HB, XC, SG, XL, and JX were primarily responsible for researching and the subsequent review of the research. XL and HB wrote the paper.

## Conflict of Interest Statement

The authors declare that the research was conducted in the absence of any commercial or financial relationships that could be construed as a potential conflict of interest.
